# E3 Ligase FBXW2 Is a New Therapeutic Target in Obesity and Atherosclerosis

**DOI:** 10.1002/advs.202001800

**Published:** 2020-08-26

**Authors:** Cheng Wang, Wenjing Xu, Yuelin Chao, Minglu Liang, Fengxiao Zhang, Kai Huang

**Affiliations:** ^1^ Clinic Center of Human Gene Research Union Hospital Tongji Medical College Huazhong University of Science and Technology Wuhan 430022 China; ^2^ Department of Rheumatology Union Hospital Tongji Medical College Huazhong University of Science and Technology Wuhan 430022 China; ^3^ Department of Cardiology Nanjing First Hospital Nanjing Medical University Nanjing 210029 China

**Keywords:** atherosclerosis, FBXW2, inflammation, insulin resistance, KSRP, obesity

## Abstract

Chronic low‐grade inflammation orchestrated by macrophages plays a critical role in metabolic chronic diseases, like obesity and atherosclerosis. However, the underlying mechanism remains to be elucidated. Here, the E3 ubiquitin ligase F‐box/WD Repeat‐Containing Protein 2 (FBXW2), the substrate‐binding subunit of E3 ubiquitin ligase SCF (a complex of FBXW2, SKP1, and cullin‐1), as an inflammatory mediator in macrophages, is identified. Myeloid‐specific FBXW2 gene deficiency improves both obesity‐associated with insulin resistance and atherosclerosis in murine models. The beneficial effects by FBXW2 knockout are accompanied by decreased proinflammatory responses and macrophage infiltration in the microenvironment. Mechanistically, it is identified that KH‐type splicing regulatory protein (KSRP) is a new bona fide ubiquitin substrate of SCF^FBXW2^. Inhibition of KSRP prevents FBXW2‐deficient macrophages from exerting a protective effect on inflammatory reactions, insulin resistance and plaque formation. Furthermore, it is demonstrated that the C‐terminus (P3) of FBXW2 competitively ablates the function of FBXW2 in KSRP degradation and serves as an effective inhibitor of obesity and atherogenesis progression. Thus, the data strongly suggest that SCF^FBXW2^ is an important mediator in the context of metabolic diseases. The development of FBXW2 (P3)‐mimicking inhibitors and small‐molecular drugs specifically abrogating KSRP ubiquitination‐dependent inflammatory responses are viable approaches for obesity and atherosclerosis treatment.

## Graphical abstract

This work demonstrates the essential role of myeloid E3 ubiquitin ligase F‐box/WD Repeat‐Containing Protein 2 (FBXW2) in obesity‐related insulin resistance and atherosclerosis. Under overnutrition exposure, FBXW2 directly interacts with and ubiquitinates KH‐type splicing regulatory protein (KSRP) in macrophages, thereby overactivating the proinflammatory reaction. A small peptide segment in the C‐terminus (P3) of FBXW2 effectively reverses chronic metabolic diseases progression.

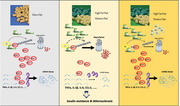

## 1 Introduction

Chronic low‐grade inflammation is involved in multiple metabolic diseases and is characterized by increased infiltration of cells from the immune system (macrophages, neutrophils, and lymphocytes) into multiple organs, such as adipose tissues, the liver, skeletal muscles and the artery wall.^[^ ^1^, ^2^, ^3^ ^]^ Several lines of evidence now support macrophages as important immune cells that orchestrate chronic inflammatory responses.^[^ ^4^, ^5^ ^]^ These chronic accumulating macrophages express inflammatory markers and secrete proinflammatory chemokines and cytokines, including tumor necrosis factor‐*α* (TNF*α*), C—C motif ligand‐2 (CCL2), interleukin 1*β* (IL‐1*β*), and interleukin 6 (IL‐6), thus disrupting the functions of multiple cells and metabolic homeostasis.^[^ ^6^, ^7^ ^]^ Therefore, targeting this chronic inflammatory aspect of macrophage biology is a therapeutic strategy to control the metabolic homoeostasis in the diseased microenvironment.

Obesity‐induced insulin resistance and atherosclerosis are inflammatory disease processes. Emerging evidence has revealed the adverse effects of inflammation in visceral fat such as decreased fatty acid and glucose uptake, ectopic triglyceride accumulation, and disruption of insulin signaling.^[^ ^8^, ^9^ ^]^ Likewise, the accumulation and activation of inflammatory macrophages in atherosclerotic plaques lead to plaque expansion and destabilization.^[^ ^10^ ^]^ However, the balance between inhibition and activation in macrophages to avoid inappropriate and detrimental inflammatory responses should be tightly controlled. Even if the regulation of macrophage activation has been well investigated, the underlying molecular regulatory network and therapeutic targets in the chronic inflammatory condition need to be further explored.

FBXW2 (F‐box and WD repeat domain‐containing 2) is a member of the WD40 repeat‐containing F‐box protein family and a component of SCF (complex of F‐box protein, SKP1 and CUL1)‐type ubiquitin ligase, which can recognize, bind and polyubiquitinate substrates for subsequent degradation by the proteasome.^[^ ^11^ ^]^ Recently, FBXW2 has been identified to function as a tumor suppressor.^[^ ^12^ ^]^ FBXW2 targets *β*‐catenin ubiquitination and degradation to regulate the invasion and migration of lung cancer cells.^[^ ^13^ ^]^ FBXW2 could also promote the ubiquitination and degradation of SKP2 (S phase kinase‐associated protein 2) to inhibit the survival and growth of lung cancer cells.^[^ ^12^ ^]^ In addition to its role as a master suppressor, FBXW2 has been identified to function in placental cell fusion and bone tissue regeneration.^[^ ^14^, ^15^ ^]^ However, whether and how FBXW2 functions in metabolic inflammation are poorly understood.

In this study, we investigated the role of FBXW2 in metabolic chronic inflammation, including obesity and atherosclerosis. Using two murine models of metabolic diseases, we found that FBXW2 in macrophages is an important inflammatory regulator in the progression of obesity‐related insulin resistance and atherosclerosis. The invention of FBXW2‐orchestrated negative regulation of chronic inflammation may lay the foundations for clinical strategies for metabolic inflammatory diseases.

## 2 Results

### 2.1 FBXW2 Is Upregulated in Macrophages of Obese Adipose Tissues and Atherosclerotic Plaques

**Figure 1:**
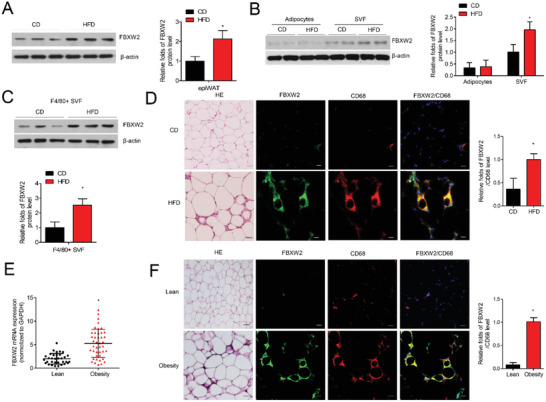
FBXW2 expression in macrophages is upregulated from obese mice and humans. Eight‐week‐old C57BL/6J mice were fed either chow diet (CD) or high fat diet (HFD) for 12 weeks. A) FBXW2 expression in epiWAT was determined by western blot assay (*n* = 8). B) Cell extracts from isolated adipocytes and SVFs in epiWAT were subject to western blot for the expression of FBXW2 (*n* = 5). C) The protein level of FBXW2 in F4/80^+^ macrophages sorted from SVFs by western blot (*n* = 5). D) Representative H&E staining of epiWAT, and the immunofluorescence images for CD68 (red) and FBXW2 (green) expression in epiWAT. Nuclei staining by DAPI (blue). Scale bars, 20 µm. E) CD14^+^ macrophages were isolated from visceral adipose tissue of lean (*n* = 33) and obese (*n* = 42) subjects. The mRNA level of FBXW2 was tested by real‐time qPCR assay. F) Representative H&E staining of visceral adipose tissues from lean and obese individuals, and the expressions of CD68 (red) and FBXW2 (green) were detected by immunofluorescence staining. Nuclei staining by DAPI (blue). Scale bars, 20 µm. The data are shown as the mean ± SEM. *p < 0.05 by Student's t test or ANOVA with the post‐hoc test.

Given the strong links among obesity, inflammation, and metabolic disease, it is wondering whether FBXW2 showed differential expression in obesity. We observed that HFD feeding for 12 weeks led to a significant increase in the protein level of FBXW2 in epididymal white adipose tissue (epiWAT) (**Figure** **1**A). In isolated adipocytes and stromal vascular fractions (SVFs) from epiWAT, we reproduced this pattern of differential expression in FBXW2. In isolated SVFs, the expression of FBXW2 was dramatically higher in HFD‐fed mice than in mice fed a normal chow diet (CD) (Figure 1B). We further isolated F4/80^+^ macrophages from epiWAT SVFs, and confirmed that the upregulation of FBXW2 in obese mice was exactly from macrophages (Figure 1C). Consistently, immunofluorescence staining showed the significantly upregulated FBXW2 levels mainly localized in CD68^+^ macrophages in epiWAT (Figure 1D). We next measured FBXW2 expression in human visceral tissue from lean subjects and overweight persons. Compared with normal‐weight controls, immunofluorescence staining revealed that in the visceral adipose tissue of obese individuals, the expression of FBXW2 in CD68^+^ macrophages was substantially increased (Figure 1F). In accordance, the FBXW2 mRNA level was much higher in CD14^+^ macrophages from visceral adipose tissue of obese patients (Figure 1E).

Atherosclerosis is a chronic metabolic inflammation during the whole pathological process.^[^ ^16^ ^]^ ApoE^−/−^ mice were fed by a western diet (WD) for 10 weeks. We observed that FBXW2 expression was increased in atherosclerotic plaques (Figure S1A, Supporting Information), and was majorly located in CD68^+^ macrophages in the aortic roots (Figure S1B, Supporting Information). Furthermore, we detected the expression level of FBXW2 in the left coronary arteries from patients with coronary heart disease (CHD) or normal heart donors who were rejected for heart transplantation. As shown in Figure S1C (Supporting Information), our results found a much higher expression of FBXW2 in the coronary arteries of CHD patients than in those of donors by the real‐time qPCR assay. Immunofluorescence staining demonstrated that FBXW2 colocalized with CD68^+^ macrophages was increased in the lesions from the CHD group (Figure S1D, Supporting Information).

These results indicated that the increased expression of FBXW2 in the macrophages of adipose tissues and aortic plaques was highly interrelated with obesity and atherosclerosis in both mice and humans, implying that macrophage FBXW2 is highly implicated in metabolic disease‐associated pro‐inflammatory response.

### 2.2 Myeloid FBXW2 Deficiency Alleviates Obesity and Metabolic Disorders

**Figure 2:**
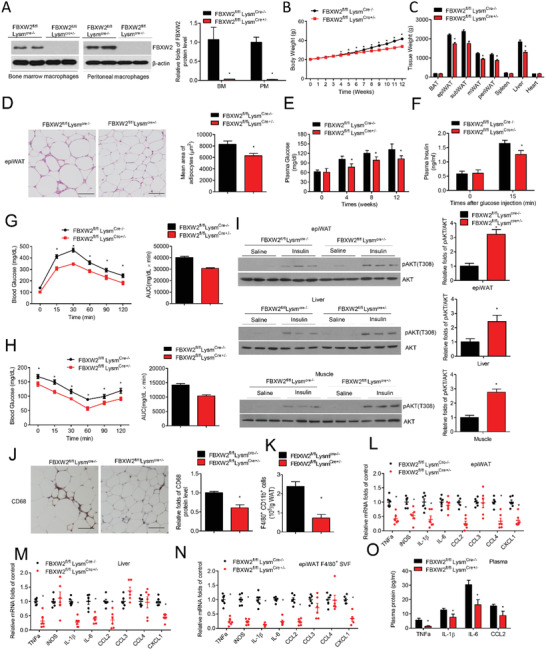
Myeloid FBXW2 deletion alleviates insulin resistance and inflammation in obesity. Age‐matched *FBXW2^fl/fl^Lysm^Cre‐/−^* and *FBXW2^fl/fl^Lysm^Cre+/−^* mice were fed a HFD for 12 weeks (*n* = 12). A) FBXW2 expression in BMDMs and PMs in *FBXW2^fl/fl^Lysm^Cre‐/−^* and *FBXW2^fl/fl^Lysm^Cre+/−^* mice were determined by western blot assay. B) Body weight was tested in HFD‐fed *FBXW2^fl/fl^Lysm^Cre‐/−^* and *FBXW2^fl/fl^Lysm^Cre+/−^* mice during the feeding time (*n* = 12). C) The different tissue weights were assessed in *FBXW2^fl/fl^Lysm^Cre‐/−^* and *FBXW2^fl/fl^Lysm^Cre+/−^* mice on HFD after 12 weeks (*n* = 12). D) H&E staining analysis and quantification of the adipocyte size of epiWAT from *FBXW2^fl/fl^Lysm^Cre‐/−^* and *FBXW2^fl/fl^Lysm^Cre+/−^* mice. Scale bars, 50 µm. E) The blood glucose in fasted *FBXW2^fl/fl^Lysm^Cre‐/−^* and *FBXW2^fl/fl^Lysm^Cre+/−^* mice (*n* = 9). F) The insulin levels in basal and stimulated condition in *FBXW2^fl/fl^Lysm^Cre‐/−^* and *FBXW2^fl/fl^Lysm^Cre+/−^* mice (*n* = 7). G) Glucose tolerance test (GTT) and H) insulin tolerance test (ITT) on fasted *FBXW2^fl/fl^Lysm^Cre‐/−^* and *FBXW2^fl/fl^Lysm^Cre+/−^* mice (*n* = 10). I) After insulin administration, tissues extracts in epiWAT, liver and skeletal muscle were subject to western bolt for the levels of AKT phosphorylation and total AKT. J) Representative immunohistochemical staining for CD68^+^ macrophages in epiWAT from *FBXW2^fl/fl^Lysm^Cre‐/−^* and *FBXW2^fl/fl^Lysm^Cre+/−^* mice (*n* = 5). Scale bars, 50 µm. K) Quantification of CD11b^+^ F4/80^+^ cells in epiWAT from *FBXW2^fl/fl^Lysm^Cre‐/−^* and *FBXW2^fl/fl^ Lysm^Cre+/−^* mice by flow cytometry (*n* = 6). The mRNA levels of proinflammatory factors in L) epiWAT, M) liver, and N) F4/80^+^ SVFs in *FBXW2^fl/fl^Lysm^Cre‐/−^* and *FBXW2^fl/fl^Lysm^Cre+/−^* mice (*n* = 6). O) Plasma contents of CCL2, TNF‐*α*, IL‐6, and IL‐1*β* in *FBXW2^fl/fl^Lysm^Cre‐/−^* and *FBXW2^fl/fl^Lysm^Cre+/−^* mice (*n* = 8). The data are shown as the mean ± SEM. *p < 0.05 by Student's *t* test.

To investigate whether FBXW2 deficiency in macrophages exerted metabolic actions, we deleted the myeloid FBXW2 gene in mice that were fed either a chow diet or a high‐fat diet for 12 weeks together with sex‐ and age‐matched littermates. FBXW2 deficiency did not alter the body weight and glucose and lipid profiles under CD‐fed conditions (**Figure** **2**A and Figure S3, Supporting Information). However, following HFD challenge, *FBXW2^fl/fl^ Lysm^Cre+/−^* mice gained less weight (Figure 2B) and displayed a lower weight in epididymal, mesenteric, perirenal and subcutaneous WAT and liver tissue than *FBXW2^fl/fl^* counterparts (Figure 2C). A smaller adipocyte size was also detected in HFD‐fed *FBXW2^fl/fl^ Lysm^Cre+/−^* mice (Figure 2D).

Obesity usually damages whole glucose metabolism in the body. As shown in Figure 2E, our data showed that FBXW2 deficiency alleviated HFD‐induced hyperglycemia and related hyperinsulinemia (Figure 2F). Furthermore, HFD‐fed *FBXW2^fl/fl^Lysm^Cre+/−^* mice were improved in disrupted glucose tolerance and insulin sensitivity (Figure 2G,H) and showed an increased readout of intracellular insulin signaling via the phosphorylation of AKT (Thr308) in epiWAT, the liver, and skeletal muscle (Figure 2I). However, HFD‐induced hypertriglyceridemia and hypercholesterolemia were not altered in *FBXW2^fl/fl^Lysm^Cre+/−^* mice (Figure S3, Supporting Information). These results demonstrate that myeloid FBXW2 deficiency may protect obesity‐induced insulin resistance.

To further investigate the underlying reasons of the positive effects of FBXW2 deficiency on metabolic disorders, we analyzed the inflammation condition in HFD‐fed myeloid FBXW2 deficiency mice. Immunohistochemistry (IHC) staining showed that less CD68^+^ macrophages accumulated in epiWAT of HFD‐fed *FBXW2^fl/fl^Lysm^Cre+/−^* mice compared with those in the control group (Figure 2J). Similarly, fluorescence‐activated cell sorting (FACS) measurements showed a decrease in macrophages infiltration in adipose tissues from obese *FBXW2^fl/fl^Lysm^Cre+/−^* mice (Figure 2K). Pro‐inflammatory chemokines and cytokines like TNF‐*α*, IL‐1*β*, CCL2 and CXCL1 were all downregulated in the epiWAT and livers of HFD‐fed *FBXW2^fl/fl^Lysm^Cre+/−^* mice (Figure 2L,M). The plasma levels of IL‐1*β*, IL‐6, TNF‐*α* and CCL2 were also obviously lower in *FBXW2^fl/fl^Lysm^Cre+/−^* mice than in *FBXW2^fl/fl^* mice (Figure 2O). Furthermore, in F4/80^+^ macrophages isolated from epiWAT, our data displayed that proinflammatory factors were dramatically decreased in HFD‐fed *FBXW2^fl/fl^Lysm^Cre+/−^* mice (Figure 2N), indicating that macrophages are a critical source of chronic systemic inflammation in *FBXW2^fl/fl^Lysm^Cre+/−^* mice after HFD feeding.

### 2.3 Myeloid FBXW2 Deficiency Mitigates Atherosclerosis

**Figure 3:**
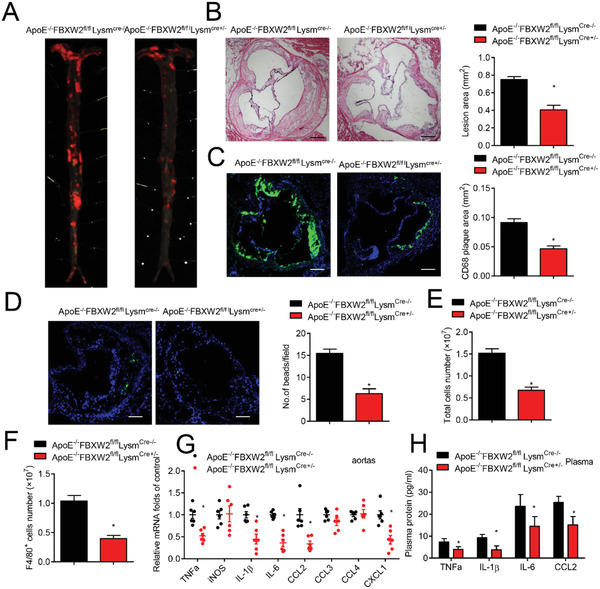
Deficiency of FBXW2 prevents atherosclerosis progression. Age‐matched *ApoE^−/−^FBXW2^fl/fl^Lysm^Cre‐/−^* and *ApoE^−/−^FBXW2^fl/fl^Lysm^Cre+/−^* mice were fed a western diet for 10 weeks (*n* = 12). A) Representative whole aortas from WD‐fed *ApoE^−/−^FBXW2^fl/fl^Lysm^Cre‐/−^* and *ApoE^−/−^FBXW2^fl/fl^Lysm^Cre+/−^* mice were stained by Oil Red O staining (*n* = 10). B) Histological analysis and quantification analysis of the lesions in aortic root from *ApoE^−/−^FBXW2^fl/fl^Lysm^Cre‐/−^* and *ApoE^−/−^FBXW2^fl/fl^Lysm^Cre+/−^* mice (*n* = 8). Scale bars, 200 µm. C) Representative immunofluorescence images for CD68 (green) in the aortic root lesions from *ApoE^−/−^FBXW2^fl/fl^Lysm^Cre‐/−^* and *ApoE^−/−^FBXW2^fl/fl^Lysm^Cre+/−^* mice, and the relative quantitation (*n* = 8). Scale bars, 200 µm. D) The images and quantitative analysis of Ly‐6C^hi^ monocytes (green) infiltrated in the lesions of *ApoE^−/−^FBXW2^fl/fl^Lysm^Cre‐/−^* and *ApoE^−/−^FBXW2^fl/fl^Lysm^Cre+/−^* mice (*n* = 6). E,F) *FBXW2^fl/fl^Lysm^Cre‐/−^* and *FBXW2^fl/fl^ Lysm^Cre+/−^* mice were intraperitoneally injected with 1 mL of 4% sterile thioglycolate media. 3 d after the injection, the total number of E) peritoneal cells and F) F4/80^+^ peritoneal cells were detected (*n* = 5). G) The transcriptional levels of proinflammatory factors in the aortas of WD‐fed *ApoE^−/−^FBXW2^fl/fl^Lysm^Cre‐/−^* and *ApoE^−/−^FBXW2^fl/fl^Lysm^Cre+/−^* mice (*n* = 6). H) Plasma contents of CCL2, IL‐6, IL‐1*β*, and TNF‐*α* in WD‐fed *ApoE^−/−^FBXW2^fl/fl^ Lysm^Cre‐/−^* and *ApoE^−/−^FBXW2^fl/fl^Lysm^Cre+/−^* mice (*n* = 8). The data are shown as the mean ± SEM. *p < 0.05 by Student's *t* test.

Chronic inflammation is also an ordinary feature of atherosclerosis. To investigate the impact of FBXW2 on the atherosclerotic progression, we crossed myeloid‐specific FBXW2 deficiency with ApoE^−/−^ mice to generate *ApoE^−/−^FBXW2^fl/fl^Lysm^Cre+/−^* mice and their littermates (*ApoE^−/−^FBXW2^fl/fl^Lysm^Cre−/−^*). After 10‐weeks western diet (WD) feeding, no potent difference in the serum lipid profiles were found between *ApoE^−/−^FBXW2^fl/fl^Lysm^Cre+/−^* mice and *ApoE^−/−^FBXW2^fl/fl^Lysm^Cre‐/−^* littermates (Figure S4, Supporting Information). However, aortic atherosclerotic lesions were decreased in *ApoE^−/−^FBXW2^fl/fl^Lysm^Cre+/−^* mice compared with those in *ApoE^−/−^FBXW2^fl/fl^Lysm^Cre‐/−^* littermates (**Figure** **3**A). *ApoE^−/−^FBXW2^fl/fl^Lysm^Cre+/−^* mice exhibited smaller lesions (Figure 3B) with fewer CD68^+^ plaque areas (Figure 3C), suggesting that FBXW2 deletion in macrophages may alleviate progression of atherosclerosis in mice.

The observation that less deposition of CD68^+^ macrophages in atherosclerotic plaques from *ApoE^−/−^FBXW2^fl/fl^Lysm^Cre+/−^* mice compelled us to explore whether FBXW2 could influence macrophage infiltration. Using fluorescent beads‐labeled pro‐inflammatory Ly‐6C^hi^ monocytes, we discovered that newly recruited monocytes were significantly decreased in *ApoE^−/−^FBXW2^fl/fl^Lysm^Cre+/−^*atherosclerotic lesions (Figure 3D). Additionally, thioglycolate‐elicited F4/80^+^ macrophages and total peritoneal cells were dramatically decreased in *FBXW2^fl/fl^Lysm^Cre+/−^* mice (Figure 3E,F). These results imply that mono‐macrophage recruitment to the artery wall may be inhibited by myeloid FBXW2 deficiency.

Macrophage accumulation in aortic tissue will initiate a pro‐inflammatory reaction.^[^ ^16^ ^]^ To confirm this activity, we analyzed the expression of many proinflammatory factors in mouse aortic lesions. The expression of TNF‐*α*, IL‐1*β*, IL‐6, and CCL2 was strongly downregulated in *ApoE^−/−^FBXW2^fl/fl^Lysm^Cre+/‐^* atherosclerotic lesions (Figure 3G). Similarly, the plasma TNF‐*α*, IL‐1*β*, IL‐6, and CCL2 levels were dramatically decreased in *ApoE^−/−^FBXW2^fl/fl^Lysm^Cre+/−^* mice (Figure 3H). Therefore, myeloid FBXW2 deficiency may extenuate inflammatory responses in the atherosclerotic artery wall. All these findings were further validated in vitro. After lipopolysaccharide (LPS) induction, FBXW2 deficiency caused significantly decreased production of proinflammatory mediators, including TNF‐*α*, IL‐1*β*, IL‐6, and CCL2, in peritoneal macrophages compared with controls (Figure S5, Supporting Information).

### 2.4 FBXW2 Interacts with KSRP

**Figure 4:**
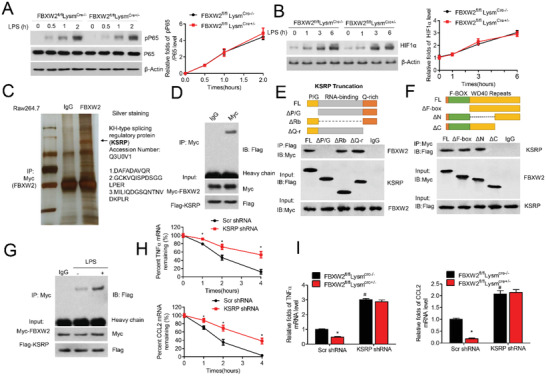
FBXW2 binds directly to KSRP. Western blot analysis of A) phosphorylated P65 (p‐P65) and total P65, and B) HIF‐1*α* in the lysates of BMDMs from *FBXW2^fl/fl^Lysm^Cre‐/−^* and *FBXW2^fl/fl^Lysm^Cre+/−^* mice with LPS stimulation for the indicated durations. C) FBXW2‐associated protein complexes from the peritoneal macrophage extracts of HFD‐fed mice were subjected to mass spectrometry analysis. The black arrow indicates the peptides identified. D) Raw264.7 cells were transfected with Myc‐tagged FBXW2 and Flag‐tagged KSRP. Co‐immunoprecipitation (Co‐IP) assays analysis of the interaction between FBXW2 and KSRP. E) Diagram of Flag‐tagged human KSRP with its domains: P/G rich domain (P/G), RNA binding domain (RBD), and Q rich domain (Q‐r). HEK293T cells were transfected with Myc‐tagged full‐length FBXW2 and Flag‐tagged KSRP mutants. Co‐IP assays demonstrated the specific binding of FBXW2 to the P/G rich domain of KSRP. F) Diagram of Myc‐tagged human FBXW2 with its domains: F‐box domain (F‐box), N‐terminal WD40 repeat domain (N), and C‐terminal WD40 repeat domain (C). 293T cells were transfected with Flag‐tagged full‐length KSRP and Myc‐tagged FBXW2 mutants. Co‐IP assays demonstrated the specific binding of KSRP to the C‐terminal WD40 repeat domain of FBXW2. G) Co‐immunoprecipitation assays analysis of the interaction between FBXW2 and KSRP after LPS (1 µg mL^−1^) treatment in BMDMs. H) Scr shRNA or KSRP shRNA lentivirus‐infected macrophages were stimulated with LPS (1 µg mL^−1^) for 12 h, followed by 5 µg mL^−1^ of ActD to block transcription. The transcriptional levels of proinflammatory factors (CCL2 and TNF‐*α*) were tested by Real‐time qPCR assay (*n* = 5). I) PMs isolated from *FBXW2^fl/fl^Lysm^Cre‐/−^* and *FBXW2^fl/fl^ Lysm^Cre+/‐^* mice were transduced with Scr shRNA or KSRP shRNA lentivirus, then treated with LPS (1 µg mL^−1^) for 12 h. The mRNA levels of proinflammatory factors (CCL2 and TNF‐*α*) were tested by Real‐time qPCR assay (*n* = 5). The data are shown as the mean ± SEM. *p < 0.05 and ^#^ *p* < 0.05 by ANOVA with the post‐hoc test.

To explore the molecular mechanisms regulating inflammatory mediator production in macrophages, we first aimed at the activation of NF‐*κ*B and HIF‐1*α* signaling pathways in bone marrow‐derived macrophages (BMDMs) during lipopolysaccharides (LPS)‐stimulated response.^[^ ^17^, ^18^ ^]^ Our results demonstrated that there was no significant difference in NF‐*κ*B and HIF‐1*α* activation between *FBXW2^fl/fl^Lysm^Cre‐/−^* and *FBXW2^fl/fl^Lysm^Cre+/−^* BMDMs (**Figure** **4**A,B), raising the possibility of other key regulators of inflammatory genes in response to inflammatory stimuli. To screen the potential mediators of FBXW2, we explored FBXW2‐interacting proteins in cellular extracts from peritoneal macrophages of HFD‐treated mice. After co‐immunoprecipitation (co‐IP), silver staining showed several additional bands in eluted proteins between the FBXW2 and IgG groups. Thereafter, the corresponding bands were subjected to an LC‐MS/MS assay. Eliminating the false positives, we focused on KH‐type splicing regulatory protein (KSRP) (Figure 4C). KSRP, an RNA binding protein that destabilizes mRNAs via adenylate/uridylate‐rich elements (AREs), has been reported to negatively modulate a subset of cytokines and chemokines by regulating mRNA stability and translational efficiency.^[^ ^19^, ^20^ ^]^ To further verify the interaction between FBXW2 and KSRP, protein extracts from Raw264.7 cells cotransfected with Myc‐FBXW2 and Flag‐KSRP were subjected to co‐IP assay, which demonstrated that FBXW2 coprecipitated with KSRP (Figure 4D). Moreover, we investigated the functional domains mediating the association of FBXW2 and KSRP. A series of KSRP deletion mutants was constructed. We observed that exogenous FBXW2 interacted directly with the P/G rich domain of KSRP (Figure 4E). Conversely, using FBXW2 deletion mutants, we mapped KSRP exclusively to the C‐terminal WD40 repeat domain of FBXW2 (Figure 4F). Thus, FBXW2 directly binds to KSRP via the P/G rich domain and C‐terminal WD40 repeat domain.

To further confirm whether KSRP was the target of FBXW2 in the modulation of pro‐inflammatory factors, we first found that the interaction between endogenous KSRP and FBXW2 in BMDMs was increased after LPS stimulation (Figure 4G). After LPS challenge, KSRP knockdown could extend the half‐life of some inflammatory factors (e.g., TNF*α*, IL‐1*β*, IL‐6 and CCL2) (Figure 4H and Figure S6, Supporting Information). More interestingly, knockdown of KSRP markedly disrupted the protective effect on the expression of some chemokines and cytokines, including CCL2, TNF‐*α*, IL‐1*β*, and IL‐6, in LPS‐treated *FBXW2^fl/fl^Lysm^Cre+/−^* macrophages (Figure 4I and Figure S6, Supporting Information). Additionally, depletion of KSRP could significantly deteriorate the mRNA degradation by the increased 3ʹ‐untranslated region (3ʹ‐UTR) luciferase activities of these genes (IL‐1*β*, IL‐6, TNF‐*α*, and CCL2), which should be declined under FBXW2 deficiency (Figure S6, Supporting Information). These results indicate that decreased inflammatory responses in FBXW2 knockdown cells are likely due to KSRP‐mediated mRNA instability.

### 2.5 FBXW2 Mediates the Ubiquitination and Degradation of KSRP

**Figure 5:**
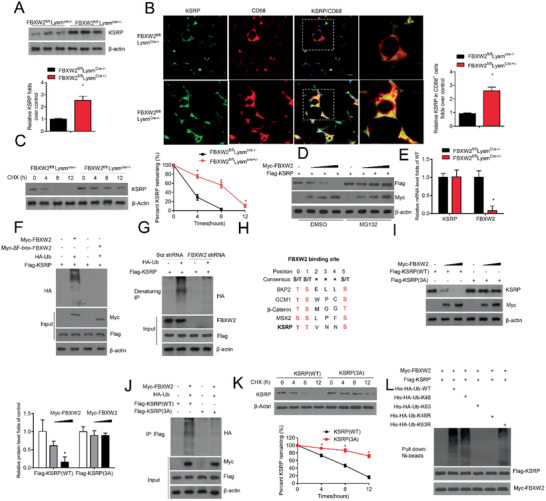
FBXW2 mediates the ubiquitination and degradation of KSRP. A) KSRP expression in *FBXW2^fl/fl^Lysm^Cre‐/−^* and *FBXW2^fl/fl^ Lysm^Cre+/‐^* peritoneal macrophages was tested by western blot assay (*n* = 5). B) Representative immunofluorescence images of KSRP and CD68 in epiWAT sections from *FBXW2^fl/fl^Lysm^Cre‐/−^* and *FBXW2^fl/fl^Lysm^Cre+/−^* mice on HFD for 12 weeks and the relative quantification (*n* = 10). Scale bars, 20 µm. C) Immunoblot analysis of the KSRP level in the lysates of *FBXW2^fl/fl^Lysm^Cre‐/−^* and *FBXW2^fl/fl^Lysm^Cre+/‐^* peritoneal macrophages treated with cycloheximide (CHX; 20 µg mL^−1^) for the indicated hours (*n* = 5). D) KSRP was coexpressed with empty vector or different contents of FBXW2 plasmids in Raw264.7 cells with or without MG132 treatment. The expression levels of KSRP and FBXW2 were analyzed using western blotting (*n* = 5). E) Real‐time qPCR assay analysis of KSRP in *FBXW2^fl/fl^Lysm^Cre‐/−^* and *FBXW2^fl/fl^Lysm^Cre+/‐^* peritoneal macrophages (*n* = 5). F) HEK293T cells were transfected with Myc‐FBXW2 or Myc‐ΔF‐box‐FBXW2 plus vectors for HA‐Ub and Flag‐KSRP and then were treated with MG132. The cell lysates were subjected to immunoprecipitation with Flag M2 beads and western blotting with anti‐HA antibody (*n* = 5). G) Raw264.7 cells were transduced with Scr shRNA or FBXW2 shRNA plus vectors for HA‐Ub and Flag‐KSRP and then were treated with MG132. The protein extracts were subjected to immunoprecipitation with Flag M2 beads and western blotting with anti‐HA antibody (*n* = 5). H) Sequence alignment of KSRP with the phospho‐degron sequences recognized by FBXW2. I) HEK293T cells cotransfected with Myc‐FBXW2 plus vectors for Flag‐KSRP (WT) or Flag‐KSRP (3A). Western blot analysis for KSRP expression in the cell lysates (*n* = 5). J) HEK293T cells were pretransfected with Myc‐FBXW2 plus Flag‐KSRP (WT), Flag‐KSRP (3A) or HA‐Ub, and then treated with MG132. Immunoprecipitation analysis for KSRP ubiquitination with anti‐Flag‐M2 beads (*n* = 5). K) Myc‐FBXW2 plus Flag‐KSRP(WT) or Flag‐KSRP (3A) were first transfected into HEK29T cells. Then cells were treated with CHX (20 µg mL^−1^) for the indicated hours. Immunoblot analysis of the KSRP levels in the cell lysates (*n* = 5). L) FBXW2 promotes KSRP ubiquitination via K48 linkage. Immunoblot analysis of His‐tag pull‐down and cell extracts derived from HEK293T cells transfected with the indicated constructs. The data are analyzed by Student's t test or ANOVA with the post‐hoc test and are presented as the mean ± SEM. *p < 0.05.

We next explored whether FBXW2 regulated the KSRP protein level. As shown in **Figure** **5**A, higher protein expression level of KSRP was observed in FBXW2‐deficient PMs (Figure 5A). Consistently, immunofluorescence (IF) staining of epiWAT from HFD‐fed *FBXW2^fl/fl^Lysm^Cre+/−^* mice showed increased KSRP expression colocalized with CD68^+^ macrophages (Figure 5B). We further explored whether FBXW2 regulated KSRP protein stability. Macrophages from *FBXW2^fl/fl^Lysm^Cre+/−^* mice showed a marked elevation in the protein abundance of endogenous KSRP due to an increase in the half‐life of endogenous KSRP (Figure 5C). Then, we examined the possibility whether FBXW2 led to KSRP downregulation via proteasome degradation. Macrophages cotransfected with Myc‐FBXW2 and Flag‐KSRP were incubated with DMSO or MG132, a potent 26S proteasome inhibitor. MG132 treatment significantly blocked the downregulation of KSRP expression in FBXW2‐overexpressed macrophages (Figure 5D). Meanwhile, we also detected the mRNA level of KSRP. Our data revealed that FBXW2 deficiency did not alter the transcriptional level of KSRP (Figure 5E). These results strongly suggest that FBXW2 targets KSRP for proteasomal degradation.

Because FBXW2 is an E3 ubiquitin ligase, its function is driven by its association and ubiquitination with other factors.^[^ ^21^ ^]^ KSRP might act as a ubiquitin substrate of FBXW2. As depicted in Figure 5F, we found that KSRP was polyubiquitinated by wild‐type FBXW2, not by the enzymatically dead mutant (ΔF‐box‐FBXW2). Additionally, the silencing of FBXW2 in Raw264.7 cells dramatically downregulated the polyubiquitination of endogenous KSRP (Figure 5G). FBXW2 often recognizes phosphorylated serine/threonine residues in the degron sequence TSXXXS, and KSRP harbors one perfectly matched FBXW2‐binding motif (Figure 5H). Thus, we generated triple mutants with all three serine/threonine residues on the recognized motif of KSRP (3A), and ubiquitination assays demonstrated that FBXW2 enhanced the polyubiquitination and degradation of KSRP(WT), but not that of the KSRP (3A) mutant (Figure 5I,J). Furthermore, the KSRP(3A) mutant prolonged the half‐life of KSRP protein (Figure 5K). Finally, we observed that FBXW2‐mediated KSRP polyubiquitination occurs via the K48 linkage for its degradation (Figure 5L). Overall, our combined results showed that KSRP is a substrate of the SCF^FBXW2^ E3 ligase, which ubiquitylates it and targets it for proteasome degradation.

### 2.6 KSRP Is Required for the Protective Effect on Inflammation Activation and Metabolic Disorders in FBXW2‐Deficient Mice

**Figure 6:**
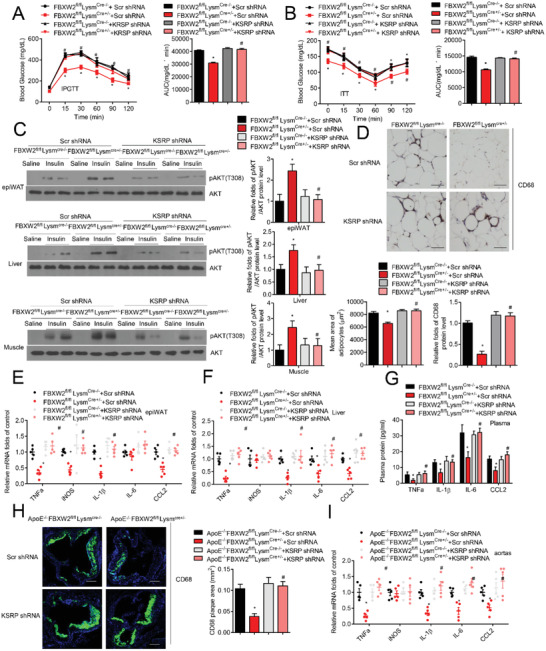
KSRP mediates the protective effects on inflammation activation, insulin resistance and atherosclerosis in FBXW2 deficiency mice. *FBXW2^fl/fl^Lysm^Cre‐/−^* or *FBXW2^fl/fl^Lysm^Cre+/−^* mice received an injection of lentivirus encoding shRNA against KSRP and then were fed with HFD for 12 weeks (*n* = 15). Fasted mice were then subjected to the A) GTT and B) ITT. C) Acute insulin signaling in epididymal fat (epiWAT), the soleus, and the liver were detected by immunoblot analysis of AKT phosphorylation. D) Representative images of CD68 immunohistochemistry in epiWAT sections. Scale bars, 50 µm. The relative quantification of the adipocyte area and CD68 level are shown. Relative mRNA levels of inflammatory factors (IL‐1*β*, iNOS, TNF*α*, CCL2 and IL‐6) in E) epiWAT and F) the liver of mice. G) The plasma concentrations of factors (IL‐1*β*, CCL2, TNF*α* and IL‐6) in the mice were tested. *ApoE^−/−^FBXW2^fl/fl^Lysm^Cre‐/−^* and *ApoE^−/−^FBXW2^fl/fl^Lysm^Cre+/−^* mice received an injection of lentivirus encoding shRNA against KSRP and then were fed a WD for 10 weeks (*n* = 15). H) Representative CD68^+^ immunofluorescence staining and quantitation in the plaques of aortic root. Scale bars, 200 µm. I) Relative mRNA levels of inflammatory mediators (TNF*α*, IL‐1*β*, iNOS, CCL2 and IL‐6) in the aortas of mice. The data are analyzed by ANOVA with the post‐hoc test and are presented as the mean ± SEM. *p < 0.05 and ^#^ *p* < 0.05.

**Figure 7:**
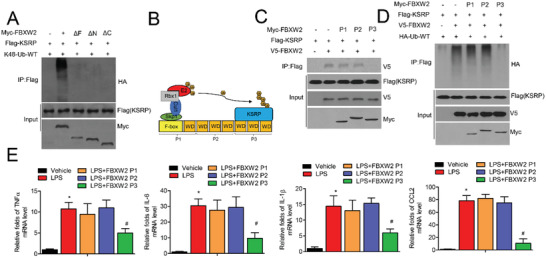
FBXW2 (P3) attenuates KSRP degradation and inflammation in vitro. A) Immunoprecipitation followed by western blot was performed to detect the KSRP ubiquitination in HEK293T cells cotransfected with Flag‐KSRP and Myc‐FBXW2 plus HA‐K48‐Ub constructs (FL, ΔC, ΔN, and ΔF). The ΔF plasmid lacks the F‐box domain. ΔC and ΔN plasmid indicate separate depletion of the C‐terminal and N‐terminal WD40 repeat regions. B) Schematic diagram of the interaction between KSRP and the SCF^Fbxw2^ complex. C) Immunoblot analysis of the V5‐FBXW2 protein after immunoprecipitation of KSRP with anti‐Flag antibody. HEK293T cells were cotransfected with Flag‐KSRP and V5‐FBXW2 in different combinations with Myc‐FBXW2 constructs (P1, 1–138 aa; P2, 139–306 aa; P3, 307–454 aa of FBXW2). D) Immunoprecipitation followed by western blot was performed to detect KSRP ubiquitination. HEK293T cells were transfected with different combinations of the indicated plasmids. E) The relative mRNA expression of proinflammatory cytokines (IL‐1*β*, TNF*α*, iNOS, CCL2, and IL‐6) was measured after LPS stimulation for 12 h in the absence or presence of the indicated segments. The data are analyzed by ANOVA with the post‐hoc test and are presented as the mean ± SEM. *p < 0.05 and ^#^ *p* < 0.05.

We next tested whether inhibition of KSRP could abrogate the beneficial effect on glucose metabolism and inflammation in HFD‐fed myeloid FBXW2‐deficient mice. HFD‐fed *FBXW2^fl/fl^Lysm^Cre‐/‐^* or *FBXW2^fl/fl^Lysm^Cre+/−^* mice received an injection of lentivirus encoding shRNA against KSRP (Figure S7, Supporting Information). Compared with HFD‐fed *FBXW2^fl/fl^Lysm^Cre+/−^* mice receiving scrambled (Scr) shRNA, KSRP knockdown in HFD‐fed FBXW2‐deficient mice markedly disrupted the alleviated body weight and glucose profile by IP‐GTT and IP‐ITT assays (Figure 7, Supporting Information, **Figure** **6**A,B). Additionally, we analyzed insulin signaling in mice on HFD. Inhibition of KSRP significantly abolished the improvement of FBXW2 deficiency in phosphorylated AKT(Thr308) level in epididymal fat, the soleus muscle, and the liver relative to that in Scr shRNA controls (Figure 6C). As expected, KSRP depletion could significantly reverse the reduced CD68‐positive macrophage infiltration and the mean area of adipocytes in epiWAT from HFD‐fed *FBXW2^fl/fl^Lysm^Cre+/−^* mice (Figure 6D). Furthermore, HFD‐fed *FBXW2^fl/fl^Lysm^Cre+/−^* mice receiving KSRP shRNA lentivirus showed a dramatical increase in the expression of some inflammatory factors, such as TNF*α*, IL‐6, IL‐1*β* and CCL2, in blood serum samples (Figure 6G). These findings were consistent with the expression of proinflammatory mediators (e.g., TNF‐*α*, IL‐1*β*, IL‐6, and CCL2) typically linked to the progression of insulin resistance in the liver and WAT (Figure 6E,F). We further verified whether KSRP was attributed to the beneficial effect on atherogenesis progression in *ApoE^−/−^FBXW2^fl/fl^Lysm^Cre+/−^* mice. As expected, depletion of KSRP could significantly reverse the lower number of CD68^+^ macrophages in atherosclerotic lesions from *ApoE^−/−^FBXW2^fl/fl^Lysm^Cre+/−^* mice (Figure 6H). Importantly, an upward trend in the pro‐inflammatory factors (TNF‐*α*, CCL2, IL‐1*β*, and IL‐6) in aortic plaques was displayed in *FBXW2^fl/fl^Lysm^Cre+/−^* mice receiving KSRP shRNA lentivirus. (Figure 6I). Thus, these data suggest that KSRP is required for the protective effect on inflammation activation and metabolic disorders from myeloid FBXW2‐deficient mice.

### 2.7 FBXW2 (P3) Competes with SCF^FBXW2^ to Suppress KSRP Degradation

To further explore which domain(s) of the FBXW2 protein is essential for KSRP degradation, we generated F‐box domain deletion, N‐terminus, and C‐terminus forms (ΔF, ΔN, and ΔC) of the FBXW2 protein (Figure 4E). All three truncations could not add K48‐linked polyubiquitin chains to KSRP (**Figure** **7**A). These findings suggest that both F‐box domain and the WD40 repeat domain of FBXW2 are required for KSRP degradation. For simplification, in the present study, the F‐box of the FBXW2 protein, which is responsible for formation of the SCF complex, is referred to as FBXW2 (P1); the C‐terminal WD40 repeat domain of the FBXW2 protein, which is critical for binding to KSRP, is referred to as FBXW2 (P3); and the N‐terminal WD40 repeat domain of the FBXW2 protein is referred to as FBXW2 (P2) (Figure 7B).

Due to the critical role of the F‐box and C‐terminal WD40 repeat domain of the FBXW2 protein in KSRP ubiquitination, we separately introduced FBXW2 (P1, P2, or P3) segments to explore their effect on the KSRP ubiquitination and inflammatory response in vitro. Surprisingly, the molecular mapping and co‐immunoprecipitation analysis indicated that only FBXW2 (P3) interacted with KSRP, thus blocking the interaction between FBXW2 and KSRP (Figure 4F,C). FBXW2 (P3), but not FBXW2 (P1) or FBXW2 (P2), blocked SCF^Fbxw2^‐induced KSRP ubiquitination (Figure 7D). Moreover, FBXW2 (P3) inhibited LPS‐induced expression of some chemokines and cytokines, including CCL2, IL‐6, IL‐1*β*, and TNF‐*α*, in macrophages (Figure 7E). Thus, FBXW2 (P3), through competing with SCF^Fbxw2^, attenuates KSRP ubiquitination and inflammatory responses in macrophages.

### 2.8 FBXW2 (P3) Mitigates Obesity and Atherosclerosis In Vivo

**Figure 8:**
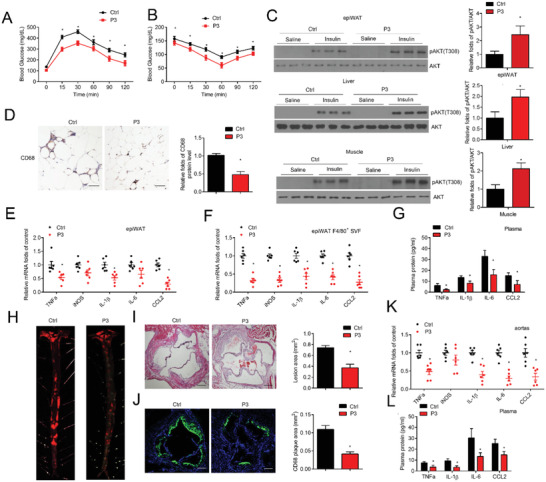
FBXW2 (P3) mitigates inflammation activation, insulin resistance and atherosclerosis in vivo. After 4 weeks of HFD feeding, the C57BL/6J mice were injected with adeno‐associated virus carrying HA‐tagged human FBXW2 (P3) to mediate FBXW2 (P3) expression in macrophages and then were fed an HFD for another 8 weeks (*n* = 15). Fasted mice were subjected to the A) GTT and B) ITT. C) Acute insulin signaling in epididymal fat (epiWAT), the soleus, and the liver were detected by immunoblot analysis of AKT phosphorylation. D) Representative images of CD68 immunohistochemistry in epiWAT sections. Scale bars, 50 µm. The relative quantification of the CD68 level is shown. Relative mRNA levels of inflammatory mediators (IL‐1*β*, TNF*α*, iNOS, CCL2 and IL‐6) in epididymal fat (epiWAT) (E) and epiWAT F40/80^+^ macrophages (F) of mice. G) Plasma concentrations of inflammatory factors (TNF*α*, IL‐1*β*, CCL2, and IL‐6) in the mice were tested. After 4 weeks of WD feeding, the ApoE^−/−^ mice were injected with adeno‐associated virus carrying HA‐tagged human FBXW2 (P3) to mediate FBXW2 (P3) expression and then were fed a WD for another 6 weeks (*n* = 15). H) Whole aortas were stained by Oil Red O staining. I) Histological analysis and J) representative CD68^+^ immunofluorescence staining of the lesions in aortic root sections, and the relative quantitation (*n* = 8). Scale bars, 200 µm. K) Relative mRNA levels of inflammatory factors (TNF*α*, IL‐1*β*, iNOS, CCL2, and IL‐6) in the aortas of mice. L) Plasma concentrations of inflammatory mediators (TNF*α*, IL‐1*β*, CCL2, and IL‐6) in the mice were tested. The data are analyzed by ANOVA with the post‐hoc test and are presented as the mean ± SEM. *p < 0.05.

Because FBXW2 (P3) acted as an inhibitor of KSRP ubiquitination and exhibited potent protective effects against LPS‐induced inflammation in vitro, we next investigated the therapeutic effects of this segment in murine models of HFD/WD‐induced obesity and atherosclerosis. The amino acid sequences of the FBXW2 (P3) are highly conserved between humans and mice based on sequence alignment. After 4 weeks of HFD/WD feeding, we delivered an adeno‐associated virus carrying HA‐tagged human FBXW2 (P3) into mouse followed by another 8/6 weeks of HFD/WD feeding. The expression of FBXW2 (P3) was measured at 12 weeks of HFD feeding. Western blotting showed that HA‐tagged FBXW2 (P3) was strikingly overexpressed in mouse peritoneal macrophages and bone marrow‐derived macrophages at the terminal point (Figure S8, Supporting Information). Overexpression of FBXW2 (P3) significantly improved the glucose profile and insulin sensitivity in HFD‐fed mice as demonstrated by IP‐GTT and IP‐ITT (**Figure** **8**A,B). We also analyzed insulin signaling in mice on HFD. FBXW2 (P3) supplement significantly upregulated the AKT (Thr308) phosphorylation stimulated by insulin in epididymal fat, the liver and soleus muscle relative to controls (Figure 8C). Meanwhile, IHC analysis revealed that the number of infiltrating CD68^+^ macrophages was obviously decreased in WAT of HFD‐fed mice receiving FBXW2 (P3) treatment (Figure 8D). We also detected the gene expression profiles of epiWAT and F4/80^+^ SVFs by qRT–PCR, and the expression levels of some inflammatory‐associated genes (CCL2, TNF*α*, IL‐6, and IL‐1*β*) were all decreased after FBXW2 (P3) treatment (Figure 8E,F). Consistently, the plasma concentrations of CCL2, IL‐1*β*, IL‐6 and TNF*α* were obviously lower in the FBXW2 (P3)‐treated group (Figure 8G). For the murine model of atherosclerosis, we found no any difference in the serum lipid profiles between FBXW2 (P3) and control‐treated ApoE^−/−^ mice (Figure S8, Supporting Information). However, the atherosclerotic plaque area in the aorta was decreased in mice after FBXW2 (P3) treatment (Figure 8H). Overexpression of FBXW2 (P3) also led to smaller lesions and a reduced CD68^+^ plaque area in aortic roots (Figure 8I,J). Additionally, western diet‐induced upregulation of pro‐inflammatory factors such as CCL2, IL‐1*β*, IL‐6, and TNF‐*α* in atherosclerotic lesions and plasma were alleviated by the overexpression of FBXW2 (P3) in vivo (Figure 8K,L). Taken together, these results strongly demonstrate that FBXW2 (P3) attenuates insulin resistance and inflammation in obesity and atherosclerosis, and could serve as a potential therapeutic strategy to treat metabolic diseases.

## 3 Discussion

Chronic inflammation is a critical process in the pathogenesis of atherosclerosis and obesity and greatly contributes to the development of chronic metabolic diseases. We demonstrated here that FBXW2 deficiency in macrophages counteracts the detrimental effects of HFD/WD on body weight, inflammation of adipose tissue, systemic insulin insensitivity and atherosclerosis. Moreover, we have uncovered FBXW2 as a component of the SCF complex that directly binds to KSRP and targets it for degradation in a ubiquitination‐dependent manner. We further discovered that FBXW2 (P3), the C‐terminal WD40 repeat domains of FBXW2, blocks the SCF^Fbxw2^‐KSRP association and KSRP degradation, thus in turn, retarding the progress of obesity and atherosclerosis. These lines of evidence strongly suggest that SCF^Fbxw2^ is an essential mediator of KSRP‐dependent proinflammatory responses in obesity‐associated metabolic disorders and that suppression of KSRP degradation is a feasible strategy to treat metabolic diseases.

Obesity leads to the development of multiple metabolic disorders.^[^ ^22^, ^23^ ^]^ In contrast, weight loss, achieved by using bypass surgery or other associated procedures, have been proven to confer effective therapeutic benefit.^[^ ^24^ ^]^ From our data, deficiency of FBXW2 in macrophage is shown to be a major origin of anti‐inflammatory and anti‐obesity signal curbing the progression of metabolic diseases. This unique feature of macrophage FBXW2 suggested it may integrate inflammatory and metabolic responses between macrophages and adipocytes. Studies have demonstrated that inflammation may act in the central nervous system as well as in the peripheral tissues to mediate energy balance.^[^ ^25^, ^26^ ^]^ Further investigation using metabolic cages and tracking mouse activity level will explore whether disruption of FBXW2 in myeloid cells influences energy expenditure, thus contributing to the early phase of weight‐loss action.

Ubiquitination has emerged as a crucial protein posttranslational modification within the cells, as it plays a critical role in maintaining multiple cellular functions.^[^ ^27^ ^]^ FBXW2, a substrate recognition component of the Skp1‐Cullin‐F‐box (SCF)‐type E3 ligase complex, balances physiological and pathological dysfunction in initiation and progression of tumor by mediating the ubiquitination and degradation of various targets, such as Skp2, GCM1, *β*‐Catenin, and MSX2.^[^ ^12^, ^21^, ^27^, ^28^ ^]^ However, our data revealed that KSRP is a new target of FBXW2. Through motif analysis and in vitro verification, we identified a perfect degron sequence of Ser/Thr residues in KSRP that is required for ubiquitination and degradation by FBXW2. Interestingly, previous studies have reported that the KLHL12 (Kelch‐Like Family Member 12)‐based CUL3 E3 ubiquitin ligase complex could ubiquitinate KSRP at the Lys109, Lys121 and Lys122 residues, causing inhibition of enterovirus internal ribosome entry site (IRES)‐mediated translation and competing against other positive IRES‐transacting factors.^[^ ^29^ ^]^ These data indicate that KSRP ubiquitination at different sites can alter protein activity by changing the topology or causing protein degradation in a proteasome‐dependent manner.

KSRP is a pivotal checkpoint of many unstable mRNAs mostly encoding pro‐inflammatory mediators. By binding to AREs in the 5ʹ‐ and 3ʹ‐untranslated regions, KSRP recruits several enzymes involved in the mRNA decay.^[^ ^30^ ^]^ KSRP‐deleted astrocytes were demonstrated to produce high levels of TNF‐*α* and IL‐1*β*, which cause a wide range of inflammatory pathologies of the central nervous system and autoimmune diseases.^[^ ^31^ ^]^ Franziska et al. also demonstrated that KSRP mediated the anti‐inflammatory response of resveratrol by directly binding to IL‐8, iNOS and TNF‐*α* mRNA.^[^ ^32^ ^]^ Similarly, in the present study, we discovered that, in macrophages, KSRP is implicated in LPS‐induced pro‐inflammatory gene expression. Importantly, FBXW2 deficiency‐mediated accumulation of KSRP dramatically disrupted the mRNA stability of several proinflammatory cytokines and chemokines, subsequently alleviating the inflammatory response and progression of metabolic diseases. Furthermore, FBXW2 (P3) attenuated FBXW2‐induced KSRP ubiquitination and ameliorated HFD‐induced obesity and atherosclerosis. These observations underscore the importance of the precise regulation of KSRP stability and related metabolic regulation in maintaining whole‐body metabolic homeostasis. Even though our recent data proposed that disrupting FBXW2‐KSRP interactions could be a way of targeting inflammation in obesity and atherosclerosis, it is expected that this may be a “general” inflammatory mechanism by which the inflammatory potential of macrophages could be regulated due to the natural characteristic. Thus, from a therapeutic and adverse event standpoint, our data expand broad implications for multiple inflammatory conditions beyond metabolic disease.

Here, we identified that fragment of FBXW2 (the WD40 repeat at the C‐terminus) can interact with KSRP and act as an inhibitor of KSRP ubiquitination mediated by the FBXW2‐containing E3 ligase. Expression of this fragment by forming nonproductive (i.e., nonubiquitinylating) in macrophages was shown to effectively ameliorate obesity and atherosclerosis. However, emerging evidences characterized FBXW2 as a tumor suppressor that inhibited survival and growth of lung cancer cells,^[^ ^12^ ^]^ it is concerning the fragment would antagonize this positive effect of FBXW2 on tumor. We suppose that FBXW2 (P3) interacts with KRSP, sealing the site that should be bound by FBXW2, so FBXW2 cannot interact with and ubiquitinate KSRP. Further investigation is still of interest to explore whether fragment (P3) affect the activity of FBXW2 WD40 domain with or without negative consequences. Considering the FBXW2 (P3) construct is at the initial stage of clinical application, another point is that other natural molecules or drugs interacting with KSRP in a manner similar to the C‐terminal domain of FBXW2 will be more therapeutically effective if they do not disrupt the mRNA stability regulatory activity of KSRP.

In conclusion, our study identified FBXW2 as a negative regulator of KSRP, which drives the mRNA instability of proinflammatory genes and leads to inflammatory responses in macrophages during obesity‐associated insulin resistance and atherosclerosis. These findings highlight the key role of FBXW2 in metabolic disease‐related chronic inflammation and suggest that macrophage FBXW2/KSRP is a novel potential strategy against metabolic diseases.

## 4 Experimental Section

##### Cell Culture

Primary BMDMs and peritoneal macrophages were performed as previously reported.^[^ ^33^, ^34^ ^]^ RAW264.7 cells and HEK293T cells were cultured as recommended by American Type Culture Collection (Rockville, MD).

##### Flow Cytometry Analysis (FACS)

Murine SVFs were isolated from epiWAT^[^ ^35^ ^]^ and then were stained with the indicated fluorescent antibodies at room temperature for 30 min. The antibodies used for FACS included CD45 (BD Biosciences, 559864), CD11b (BD Biosciences, 553310), and F4/80 (BD Biosciences, 565411) antibodies. We used Aqua L‐D (Invitrogen) to exclude dead cells. Unstained, single‐stained, and fluorescence‐minus‐one controls were used for setting compensation and gates. Data were analyzed by FlowJo Software version 7.6.4.

##### Co‐Immunoprecipitation

Co‐IP assay was performed as previously reported,^[^ ^36^, ^37^ ^]^ with antibodies specific for Myc (Cell Signaling Technology, 2276), Flag (Cell Signaling Technology; 14793). The bead bound proteins were released and analyzed by Western blot.^[^ ^38^ ^]^

##### Animal Experiments

All the animal experimental procedures were approved by the Institutional Animal Care and Use Committee of Tongji Medical College of Huazhong University of Science and Technology. *FBXW2^fl/fl^* mice (on a C57BL/6J background) were crossed with lysosome 2‐Cre mice to generate myeloid FBXW2 deficiency mice. *ApoE^−/−^* mice and *FBXW2^fl/fl^ Lysm* ^cre+/−^ mice were crossed to obtain *ApoE^−/−^FBXW2^fl/fl^ Lysm^cre+/−^* mice and *ApoE^−/−^FBXW2^fl/fl^Lysm^cre‐/−^* littermates (Figure S2, Supporting Information).

##### Insulin Tolerance Test and Glucose Tolerance Test

We performed insulin tolerance test on 4 h fasted and glucose tolerance test on 14 h fasted mice by an intraperitoneal injection of insulin (1 U kg^−1^, Sigma) and d‐glucose (1.5 g kg^−1^, Sigma). Glucose concentrations were measured with Accu‐Chek Inform Glucose Monitoring kit.

##### Histological Analysis

After fixing, embedding and cutting, tissue sections were stained with H&E for morphology evaluation.^[^ ^38^ ^]^ Immunohistochemistry and immunofluorescence staining were performed as previously reported,^[^ ^38^, ^39^ ^]^ with antibodies specific against CD68 (Bio‐Rad, MCA1957; Abcam; ab6640), FBXW2 (Invitrogen, PA5‐18189), and KSRP (Abcam, ab140648).

##### Human Tissue Samples

All the human samples procedures complied with the principles outlined in the Declaration of Helsinki. All the selected patients provided written informed consent. The study was supported by the Ethics Committee of Tongji Medical College of Huazhong University of Science and Technology.

##### Statistical Analysis

The data were presented as the mean ± SEM, and the results were analyzed using GraphPad Prism (GraphPad Software Inc, San Diego). After a normal distribution was confirmed using the Kolmogorov‐Smirnov test, statistical comparisons were made using Student's *t* test for unpaired data to compare two groups and ANOVA followed by the Bonferroni post‐hoc test for multiple comparisons as appropriate. Differences with *p* < 0.05 were considered statistically significant.

## Conflict of Interest

The authors declare no conflict of interest.

## Supporting information

Supporting Information
