# Supplementary material for: E3 Ligase FBXW2 Is a New Therapeutic Target in Obesity and Atherosclerosis
Source: Adv Sci (Weinh). 2020 Aug 26;7(20):2001800. doi: 10.1002/advs.202001800 (PMC7578860; doi:10.1002/advs.202001800)
Supplement: Supplementary file 1 — Supporting Information [file ADVS-7-2001800-s001.pdf]

© 2020 Wiley-VCH GmbH

ADVANCED SCIENCE
Open Access

Supporting Information

for *Adv. Sci.,* DOI: 10.1002/advs.202001800

E3 Ligase FBXW2 Is a New Therapeutic Target in Obesity and Atherosclerosis

Cheng Wang, Wenjing Xu, Yuelin Chao, Minglu Liang, Fengxiao Zhang, and Kai Huang**

Detailed Experimental Section

Animal Experiments

FBXW2$^{fl/fl}$ mice (on a C57BL/6J background) were crossed with lysosome 2-Cre mice to generate myeloid-specific FBXW2 deficiency mice. *ApoE*$^{-/-}$ mice and *FBXW2*$^{fl/fl}$ *Lysm*$^{cre+/-}$ mice were then crossbred to obtain *ApoE*$^{-/-}$*FBXW2*$^{fl/fl}$ *Lysm*$^{cre+/-}$ mice and *ApoE*$^{-/-}$ *FBXW2*$^{fl/fl}$ *Lysm*$^{cre-/-}$ littermates (Supplementary Figure 2). All the animals were maintained under controlled temperature (22.5℃) and illumination (12-h. dark/light cycle). All the experimental procedures were approved by the Institutional Animal Care and Use Committee of Tongji Medical College of Huazhong University of Science and Technology.

For the HFD-induced obesity model, myeloid FBXW2 deficiency mice (8 weeks of age) and age-matched littermates were fed ad libitum either a CD or a 60% HFD (D12492; Research Diets) for 12 weeks. For atherosclerosis experiments, eight-week-old *ApoE*$^{-/-}$*FBXW2*$^{fl/fl}$ *Lysm*$^{cre+/-}$ mice and *ApoE*$^{-/-}$ *FBXW2*$^{fl/fl}$ *Lysm*$^{cr-/-}$ littermates were administered a western diet (WD; 15.8% fat and 1.25% cholesterol) or normal chow (NC) for 10 weeks.

Acute tissue insulin signaling tests

Overnight-fasted male *FBXW2*$^{fl/fl}$*Lysm*$^{Cre-/-}$ and *FBXW2*$^{fl/fl}$*Lysm*$^{Cre+/-}$ mice after HFD for 12 weeks were anesthetized with a muscular injection of ketamine/xylazine, followed by portal vein injection of human insulin (0.5 U/kg body weight) or vehicle saline. After 5 minutes, the epididymal fat pads, liver and soleus muscle were collected to determine the phosphorylation of AKT (Thr308), total AKT by western blotting.

Intraperitoneal glucose and insulin tolerance tests

Following a 14-h fast, the mice were intraperitoneally injected with glucose (1.5 g/kg) and blood samples for glucose measurement were collected from the tail vein at the indicated times. After a 4-h fast, insulin tolerance was assessed by the intraperitoneal injection of human regular insulin (1 U/kg). Glycemia was assessed using the Accu-Chek® Inform Glucose Monitoring kit. The plasma insulin level was analyzed using an insulin ELISA kit (Mercodia, Sweden).

Biochemical Analysis

The plasma levels of mouse IL-1β (MLB00C, R&D Systems), IL-6 (431304, BioLegend), TNFα (430904, BioLegend) or CCL2 (MJE00, eBioscience) were measured using ELISA kits. Total triglycerides and cholesterol in mouse serum were detected using the Infinity™ Triglycerides Liquid Stable Reagent and Cholesterol Liquid Stable Reagent (Thermo), respectively.

Quantification of the atherosclerosis burden

For en face analysis, the whole aorta was opened longitudinally, stained with Oil Red O for 10 min, and then was photographed with a Canon camera. The percentage of the Oil Red O-positive staining area was analyzed and quantified using ImagePro Plus software. For cross-sectional analysis of the lesion areas in the aortic root, 8-μm-thick sections throughout the aortic sinus were obtained for calculation using ImagePro Plus software.

Monocyte recruitment assays

Experimental male mice were fed a WD for 10 weeks. Clodronate liposomes (250 μl; Liposoma) were i.v. injected into transiently depleted monocytes. After 48 h, the mice were administered by i.v. injection 300 μl of fluorescence microspheres (2.5% solids [w/v]; Polysciences). The mice were euthanized, and then 8 μm-thick serial cryosections of the hearts were prepared from the atrioventricular valve. Images were then captured using a fluorescence microscope (Carl Zeiss).

Western blot assay

Western blotting was performed as described previously[1, 2]. The tissues and cells were homogenized and separated by SDS-PAGE and then were transferred to nitrocellulose membranes. The blots were probed with the appropriate primary antibodies against FBXW2 (Abcam, ab83467; Proteintech, #11499-1-AP; Invitrogen, PA5-18189), KSRP (Cell Signaling Technology; 13395), HIF1α (Cell Signaling Technology; 36169), pP65 (Cell Signaling Technology; 3033), total P65 (Cell Signaling Technology; 8242), pAKT (Cell Signaling Technology; 13038), total AKT (Cell Signaling Technology; 9272), HA (Cell Signaling Technology; 3724), Myc (Cell

Signaling Technology, 2276), Flag (Cell Signaling Technology; 14793), V5 (Cell Signaling Technology; 80076), and β-Actin (Santa Cruz; sc-47778) at 4 °C overnight. The membrane was extensively washed and then incubated with the appropriate secondary antibody. The signals were captured with Image Lab software.

RNA Isolation and Quantitative real-time PCR (qRT-PCR)

Total RNA was extracted from frozen tissue or cultured cells using TRIzol reagent (Invitrogen). Reverse transcription was performed with 1 μg of total RNA using the iScript cDNA Synthesis Kit (Bio-Rad, CA) according to the manufacturer's protocol. PCR amplification was performed using the SYBR PCR mix (Takara, Japan). The relative RNA amount was calculated using the $2\text{-}^{\Delta\Delta}$Ct method and normalized with 18S RNA.

Flow cytometry analysis

Murine SVFs were isolated from epiWAT[3] and then were resuspended in phosphate-buffered saline (PBS) supplemented with 0.5% fetal bovine serum (FBS) and stained with the indicated fluorescent antibodies for 30 min at room temperature. The antibodies used for FACS included CD45-APC (BD Biosciences, 559864), CD11b-FITC (BD Biosciences, 553310) and F4/80-BV421 (BD Biosciences, 565411) antibodies. We used Aqua L-D (Invitrogen) to exclude dead cells. Unstained, single-stained and fluorescence-minus-one controls were used for setting compensation and gates. Cells were then subjected to flow cytometry analysis with a BD FACSAria IIu flow cytometer (BD Bioscience). Data were analyzed with FlowJo Software version 7.6.4.

Immunohistochemistry staining

Epididymal fat tissues were excised, fixed and embedded in paraffin and sectioned. The slides were stained with anti-CD68 antibody (Abcam; ab6640) overnight at 4°C and then were incubated with labeled peroxidase-secondary antibodies followed by DAB+ chromogen detection (DAKO). After final washing, the sections were counterstained with hematoxylin and then were mounted and visualized using an OLYMPUS BX53 microscope.

Immunofluorescence staining

The tissue slides or cells were incubated with antibodies specific against CD68 (Bio-Rad; MCA1957), FBXW2 (Invitrogen, PA5-18189), and KSRP (Abcam, ab140648) overnight at 4°C. The samples were then rinsed and incubated with fluorescence-labeled secondary antibodies for 1 h. The nucleus was stained with DAPI. The slides were mounted and visualized using an OLYMPUS BX53 microscope.

Cell culture

Peritoneal macrophages were prepared using peritoneal lavage with 10 ml of PBS with 10% FBS. Macrophages were plated at 1.0×10^6 per ml of Dulbecco's Modified Eagle's Medium (DMEM) containing 10% FBS. After 2 h of incubation at 37 °C, the non-adherent cells were removed by washing with PBS and adherent cells were collected for subsequent experiments. Primary BMDMs were collected by flushing the bone marrow of the murine femur and tibia. The cells were cultured in DMEM medium supplemented with mouse macrophage colony-stimulating factor (M-CSF) recombinant protein for 7 days. RAW264.7 cells and HEK293T cells (ATCC, USA) were cultured with DMEM medium containing 10% FBS and were maintained at 37 °C in 5% CO_2 and 95% air.

Isolation of adipose tissue macrophages

Epididymal fat pads were excised and minced in PBS containing 0.5% BSA. Tissue suspensions were centrifuged to remove free leukocytes and erythrocytes. Collagenase D (Roche, 11088874103) was added to 1 mg/ml and was incubated at 37°C for 40 min with shaking. The cell suspension was filtered using a 100-µm filter and subjected to magnetic immunoaffinity isolation with anti-F4/80 antibodies conjugated to magnetic beads (MACS; Miltenyi Biotec). The cells were isolated using positive-selection columns before the preparation of whole-cell lysates for mRNA analysis.

Coimmunoprecipitation

Briefly, 500 µg of protein extracts was incubated with the indicated antibodies or nonspecific IgG at 4°C overnight, and protein-A/G agarose was added for another 3 hours at 4°C. The immunoprecipitants were pelleted by centrifugation and washed

with RIPA lysis buffer. The pellets were then suspended in SDS loading buffer and then were subjected to western blot assays.

LC-MS/MS analysis of FBXW2-interacted proteins

For LC-MS/MS analysis of FBXW2-interacted proteins, protein extracts from murine peritoneal macrophages after HFD treatment were mixed with anti-FBXW2 antibody or IgG antibody, followed by incubation overnight at 4 °C with rotation. Protein-A/G agarose beads were added, followed by additional incubation for 3 hours at 4 °C. After washing with RIPA lysis buffer, the immunoprecipitated proteins were subjected to western blotting and silver staining. The bands of interest were then subjected to LC-MS/MS analysis. The data were analyzed using Protein Pilot software (AB SCIEX, USA).

Plasmid construction

PCR-amplified segments of full-length and truncated human FBXW2 or KSRP were inserted into pcDNA5 vectors or pGEX-6p-1 vectors as indicated. A series of variant KSRP constructs with serine/threonine mutated to alanine was obtained according to standard molecular biology techniques. FBXW2 (P3) was amplified and inserted into pAAV CD68-mCherry (Addgene, 75033) to generate FBXW2 (P3) adeno-associated viruses under a CD68 promoter[4]. WT and the derivative Ub were gifts from Dr. Hongbing Shu (Wuhan University).

Luciferase Activity Assay

The 3′untranslated region (UTR) sequences of IL-1β, IL-6, TNFα and CCL2 were inserted into the pMIR-REPORT vector (Ambion, USA). Raw264.7 cells or 293T cells were co-transfected with the plasmids or FBXW2 siRNA according to the manufacturer’s instructions. The luciferase activities were measured using the Dual-Luciferase Reporter Assay System (Promega, USA).

Analysis of mRNA decay

Peritoneal macrophages were cultured in the presence of the transcriptional inhibitor actinomycin D (ActD, 5 ug/ml; MilliporeSigma) for various durations (0, 1, 2, 4 h) to study the rate of decay of the IL-1β, IL-6, TNFα and CCL2 mRNA.

Analysis of Human Samples

All the procedures involving human samples complied with the principles outlined in the Declaration of Helsinki. The study was supported by the Ethics Committee of Tongji Medical College of Huazhong University of Science and Technology, and all the selected patients provided written informed consent. Donors were classified as lean or obese based on the BMI following the World Health Organization criteria. For human adipose samples, visceral adipose tissue samples were obtained from gender- and age-matched human donors undergoing non-acute surgical interventions, such as hernia or cholecystectomy, in a scheduled routine surgery: control n = 33 (BMI 21.13 ± 1.97); obesity n = 42 (BMI 32.19 ± 3.53). For human plaque samples, atherosclerotic plaques from patients with CHD (coronary heart disease) undergoing heart transplants at our institution were fixed with paraformaldehyde and embedded in paraffin for histological analysis.

To assess the expression of FBXW2, paraffin sections were stained with an FBXW2 antibody (Invitrogen; PA5-64343). To isolate SVFs, human adipose tissue samples were digested using collagenase type II (1.5 mg/ml; Sigma) at 37 °C for 30 min. All the cell suspensions were filtered through a 200-μm cell strainer, followed by centrifugation at 1000 × g for 10 min, and incubation of the pellet containing the SVFs with red blood cell lysis buffer. The SVFs were resuspended in PBS supplemented with 1% FBS. $CD14^+$ macrophages were purified using magnetic beads (BD Biosciences) according to the manufacturer's instructions. Finally, the cells were prepared for mRNA analysis.

Reference

1 C. Wang, F. Zhang, L. Wang, Y. Zhang, X. Li, K. Huang, M. Du, F. Liu, S. Huang, Y. Guan, and D. Huang, *Mol Cell Biol*. 33, 4492-503 (2013)

2 C. Wang, W. Xu, J. An, M. Liang, Y. Li, F. Zhang, Q. Tong, and K. Huang, *Nat Commun*.

10, 1203 (2019)

3 E. Dalmas, A. Toubal, F. Alzaid, K. Blazek, H. L. Eames, K. Lebozec, M. Pini, I. Hainault, E. Montastier, R. G. Denis, P. Ancel, A. Lacombe, Y. Ling, O. Allatif, C. Cruciani-Guglielmacci, S. Andre, N. Viguerie, C. Poitou, V. Stich, A. Torcivia, F. Foufelle, S. Luquet, J. Aron-Wisnewsky, D. Langin, K. Clement, I. A. Udalova, and N. Venteclef, *Nat Med.* 21, 610-8 (2015)

4 P. M. Grace, K. A. Strand, E. L. Galer, D. J. Urban, X. Wang, M. V. Baratta, T. J. Fabisiak, N. D. Anderson, K. Cheng, L. I. Greene, D. Berkelhammer, Y. Zhang, A. L. Ellis, H. H. Yin, S. Campeau, K. C. Rice, B. L. Roth, S. F. Maier, and L. R. Watkins, *Proc Natl Acad Sci U S A.* 113, E3441-50 (2016)

Supplementary Figure

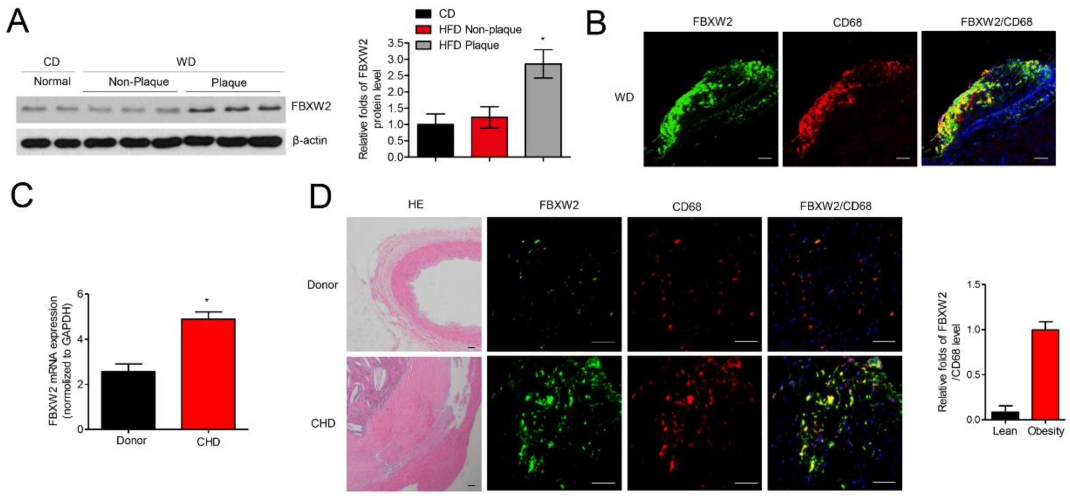

Supplementary Figure 1 FBXW2 expression is upregulated in macrophages in atherosclerosis. ApoE$^{-/-}$ mice were fed a CD or WD for 10 weeks. (A) Western blot analysis of FBXW2 expression in murine aortas (n=5). (B) Immunofluorescence staining of CD68 (red), FBXW2 (green) and their co-localization (yellow merge) in

atherosclerotic plaques. Nuclei were stained with DAPI (blue). Scale bars, 20 μm. (C) The mRNA level of FBXW2 in the coronary arteries of healthy donors and patients with CHD (n=8). (D) Representative images showing double-immunofluorescence staining for FBXW2 (green) and CD68 (red) in the coronary arteries of normal donors and patients with CHD (n=5). Scale bar=50μm. Data are expressed as mean ± SEM. *P < 0.05 by Student's t test or ANOVA with post hoc test.

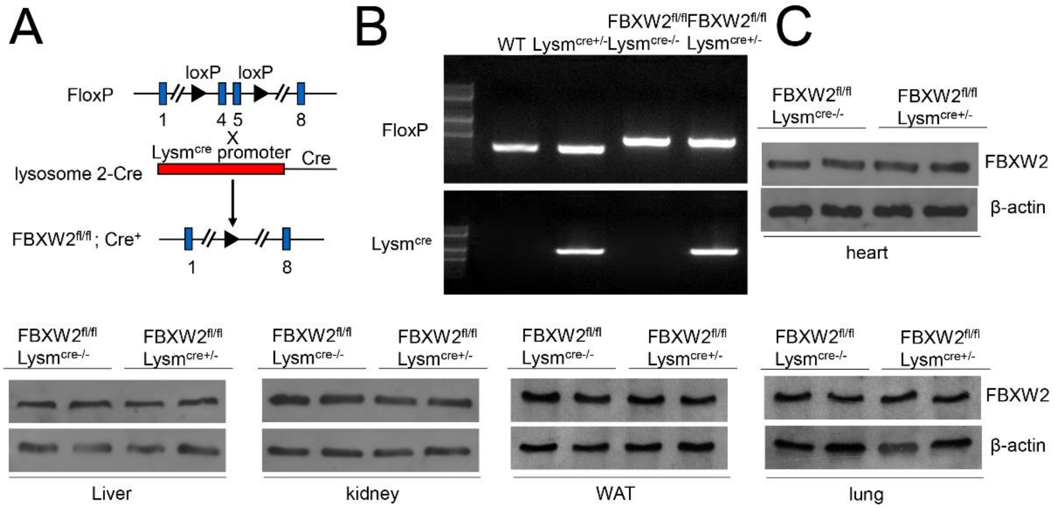

Supplementary Figure 2 Establishment of myeloid-specific FBXW2 deficiency mice. (A) Schematic work flow of the establishment of FBXW2 loxp/loxp mouse strain. (B) Representative genotyping PCR amplification of WT, Lysm-Cre, $FBXW2^{fl/fl}Lysm^{Cre-/-}$ and $FBXW2^{fl/fl}Lysm^{Cre+/-}$ mice. (C) Representative western blot analysis of FBXW2 in the indicated tissues from $FBXW2^{fl/fl}Lysm^{Cre-/-}$ and $FBXW2^{fl/fl}Lysm^{Cre+/-}$ mice (n = 8).

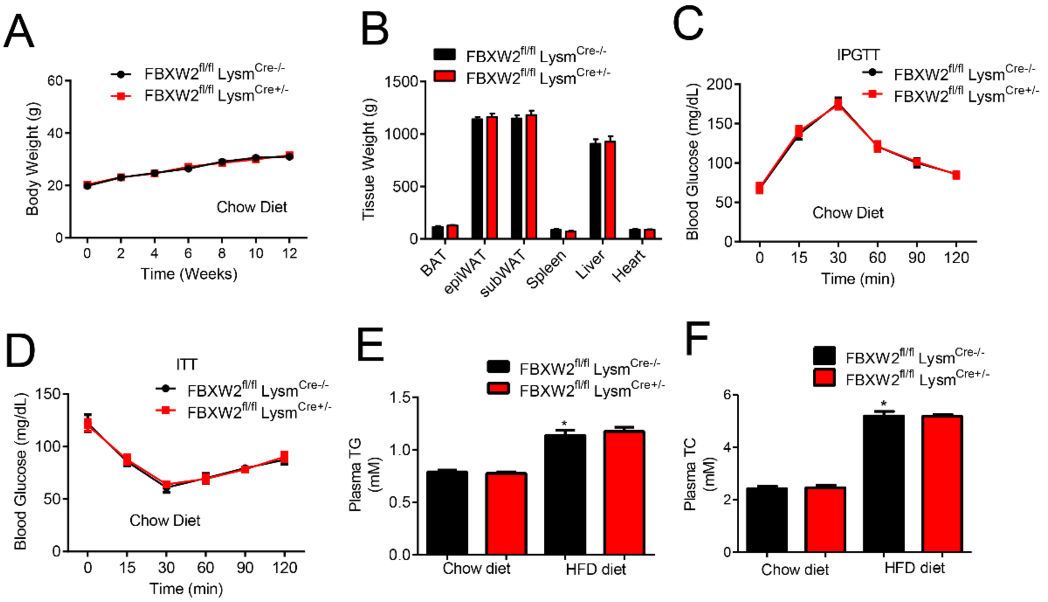

Supplementary Figure 3 Glycolipid metabolism in myeloid-specific FBXW2 deficiency mice fed a CD. $FBXW2^{fl/fl}Lysm^{Cre-/-}$ and $FBXW2^{fl/fl}Lysm^{Cre+/-}$ mice were fed a CD for 12 weeks. (A) Body weight was tested in chow diet-fed $FBXW2^{fl/fl}Lysm^{Cre-/-}$ and $FBXW2^{fl/fl}Lysm^{Cre+/-}$ mice during the feeding time (n=12). (B) The different tissue weight was tested in $FBXW2^{fl/fl}Lysm^{Cre-/-}$ and $FBXW2^{fl/fl}Lysm^{Cre+/-}$ mice on CD for 12 weeks (n=12). (C and D) GTT and ITT in $FBXW2^{fl/fl}Lysm^{Cre-/-}$ and $FBXW2^{fl/fl}Lysm^{Cre+/-}$ mice (n=5). (E) Plasma levels of TG and TC in chow diet and high fat diet-fed $FBXW2^{fl/fl}Lysm^{Cre-/-}$ and $FBXW2^{fl/fl}Lysm^{Cre+/-}$ mice (n=8). Data are expressed as mean ± SEM. *$P < 0.05$ by Student's t test or ANOVA with post hoc test.

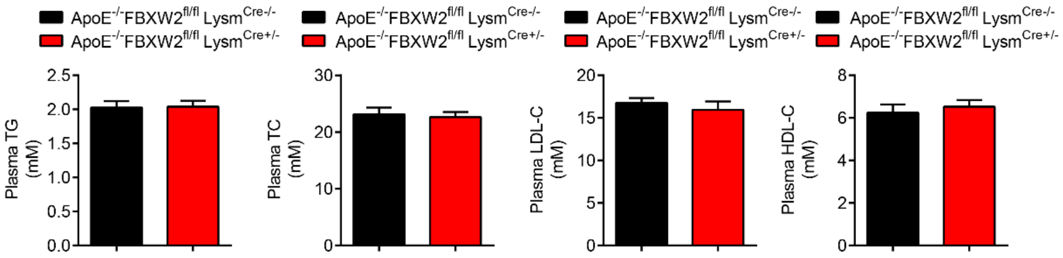

Supplementary Figure 4 Lipids profiles in *ApoE$^{-/-}$FBXW2$^{fl/fl}$Lysm$^{Cre-/-}$* and *ApoE$^{-/-}$FBXW2$^{fl/fl}$ Lysm$^{Cre+/-}$* mice after 10 weeks of WD feeding (n = 9).

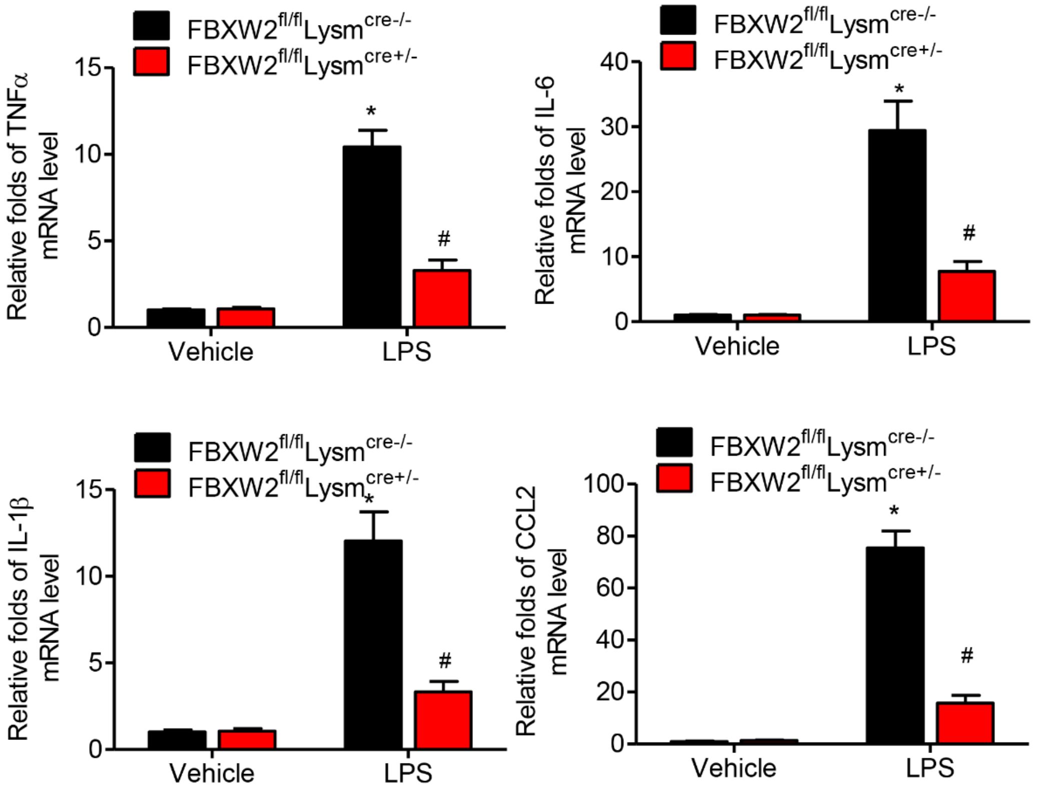

Supplementary Figure 5 PMs isolated from CD-fed *FBXW2$^{fl/fl}$Lysm$^{Cre-/-}$* and *FBXW2$^{fl/fl}$ Lysm$^{Cre+/-}$* mice were treated with LPS (1ug/ml) for 12h. The mRNA levels of inflammatory mediators (TNF-α, IL-1β, IL-6 and CCL2) were assessed by RT-qPCR (n=5). Data are expressed as mean ± SEM. $^{*}P < 0.05$ and $^{\#}P < 0.05$ by ANOVA with post hoc test.

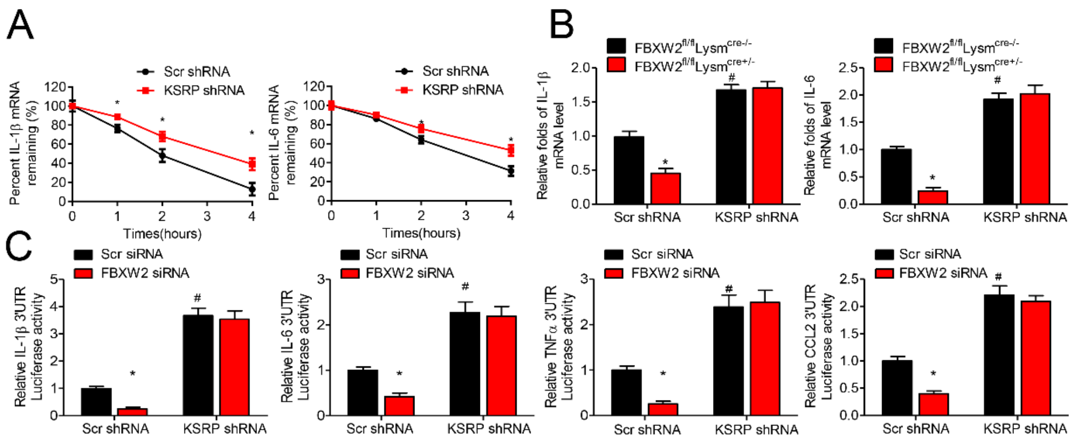

Supplementary Figure 6 (A) Macrophages pre-infected with Scr shRNA or KSRP shRNA lentivirus were treated with LPS (1 μg/mL) for 12 h, followed by 5 μg/mL ActD . The mRNA levels of inflammatory mediators (IL-1β and IL-6) were assessed by RT-qPCR. (n=5) (B) PMs isolated from *FBXW2*$^{fl/fl}$*Lysm*$^{Cre-/-}$ and *FBXW2*$^{fl/fl}$ *Lysm*$^{Cre+/-}$ mice were infected with Scr shRNA or KSRP shRNA lentivirus and then treated with LPS (1ug/ml) for 12h. The mRNA levels of inflammatory mediators (IL-1β and IL-6) were assessed by RT-qPCR (n=5). (C) Cultured 293T cells were pre-transfected with plasmids containing the 3'UTR of TNF-α, IL-1β, IL-6 or CCL2, along with FBXW2 siRNA or KSRP shRNA lentivirus for 24h. The relative luciferase activities are expressed as a percent of values determined in control group (n=5). Data are expressed as mean ± SEM. **P* < 0.05 and $^{\#}$*P* < 0.05by ANOVA with post hoc test.

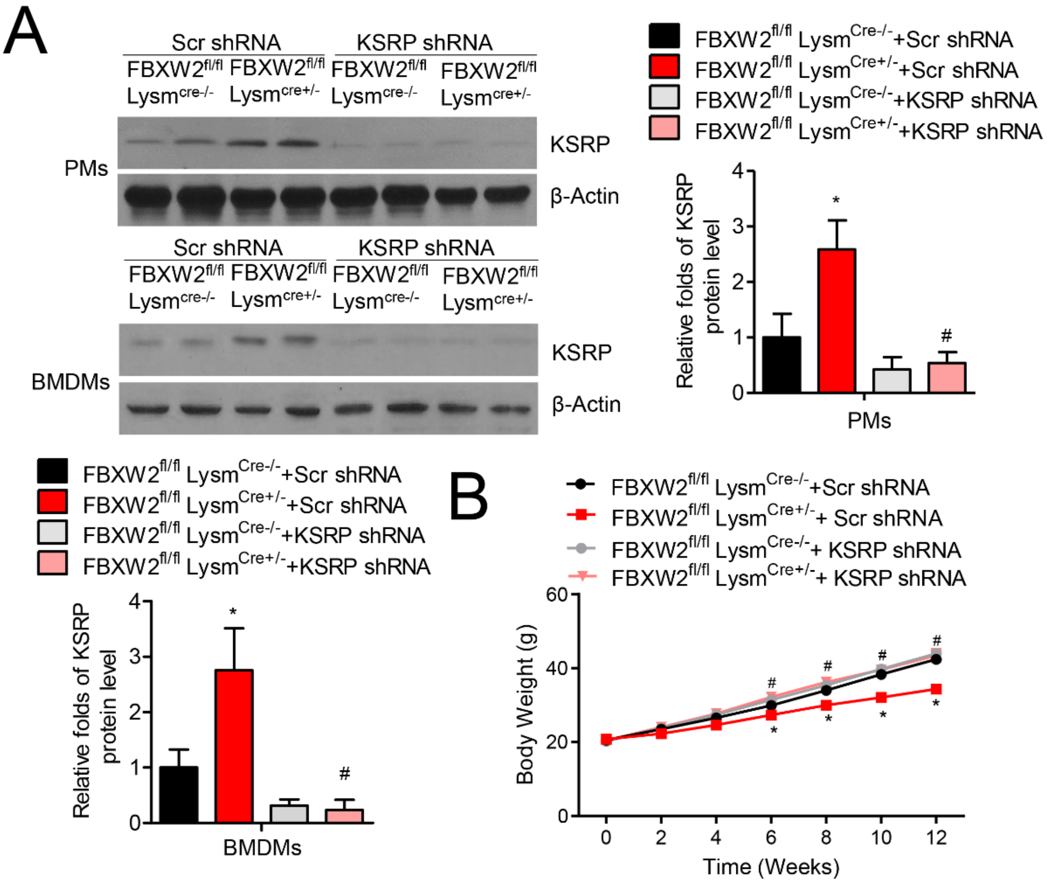

Supplementary Figure 7 *FBXW2*$^{fl/fl}$*Lysm*$^{Cre-/-}$ or *FBXW2*$^{fl/fl}$*Lysm*$^{Cre+/-}$ mice received an injection of lentivirus encoding shRNA against KSRP and then were fed with HFD for 12 weeks (n=15). (A) Western blot analysis of KSRP expression in PMs and BMDMs (n=5). (B) Body weight was tested during the feeding time (n=8). Data are expressed as mean ± SEM. *$P < 0.05$ and $^{\#}P < 0.05$ by ANOVA with post hoc test.

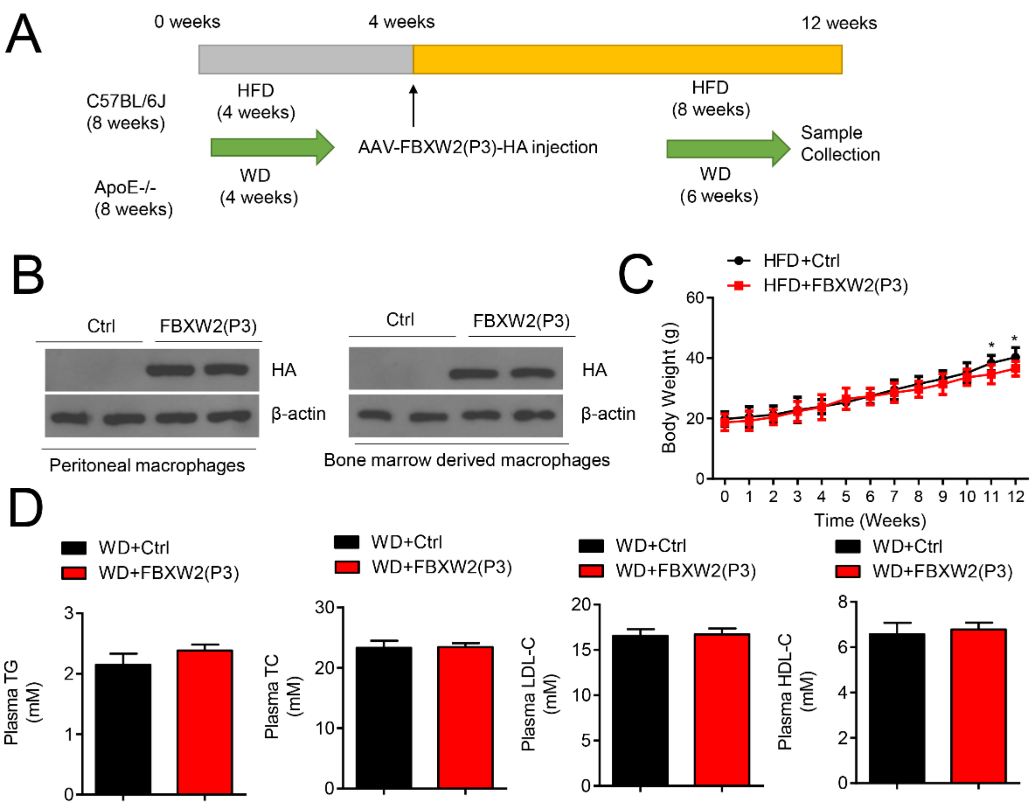

Supplementary Figure 8 (A) Schematic of the experimental procedure to test the role of FBXW2 (P3) in mice with obesity and atherosclerosis. (B) Western blot analysis of the protein levels of HA-tagged FBXW2 (P3) by an anti-HA antibody in peritoneal macrophages and bone marrow derived macrophages from the indicated mice after HFD feeding 12 weeks. (C) Body weight of the two groups of mice (n = 10). (D) Lipids profiles in three groups of mice after 10 weeks of WD feeding (n = 10). Data are expressed as mean ± SEM. **P* < 0.05 by ANOVA with post hoc test.
